# Complete Genome Sequence of a Hepatitis D Virus Genotype 1 Strain Isolated in Guangdong, China

**DOI:** 10.1128/genomeA.01505-15

**Published:** 2015-12-23

**Authors:** Baolin Liao, Weilie Chen, Qu Ping, Haiyan Shi, Haolan He, Huiyuan Liu, Yizhou Tan, Chunliang Lei, Weiping Cai

**Affiliations:** Guangzhou No. 8 People’s Hospital, Guangzhou Medical University, Guangzhou, China

## Abstract

Here, we report the first complete genome sequence of a hepatitis D virus genotype 1 strain, GZ37, isolated in Guangzhou, Guangdong Province, China, in 2014. The sequence information provided here will help us understand the molecular epidemiology of hepatitis D virus and contribute to disease control in mainland China.

## GENOME ANNOUNCEMENT

It is estimated that about 18 million people have evidence of exposure to hepatitis D virus (HDV) worldwide (1). The virus is most highly endemic in Mediterranean countries and the Middle East (2). Studies have consistently indicated that most patients with hepatitis B virus (HBV)-HDV coinfection have more severe liver disease than those with HBV monoinfection (3), while HDV genotype is associated with the severity of the disease (4–6).

To date, no complete genome sequence of the Chinese HDV strain has been reported, except for that for strain JN (GenBank accession no. HM046802). JN was isolated about 5 years ago in Shandong, which is located in northeastern China. However, Guangdong is located in southern China and is an internal and international crossroads for cultural and commercial exchanges (7). The continuous socioeconomic advancement and migration flow in Guangdong may influence the HDV genotype distribution pattern at both the local and national level. However, there have been no studies on the genotypes of HDV in Guangdong.

Here, we report the first complete genome sequence of an HDV genotype 1 (HDV-1) strain in Guangdong, GZ37, which was isolated on 13 June 2014 from a serum sample from a 32-year-old female patient with HBV-HDV coinfection. The patient was diagnosed by a positive IgM anti-HDV result (8, 9). Total RNA was extracted from the patient’s serum sample, which was stored at -80°C, using the QIAamp viral RNA minikit (Qiagen, Germany). Next, nested-reverse transcription-PCR (RT-PCR) with 4 primer pairs was used to amplify 4 overlapping amplicons spanning the entire genome. First-round PCR was conducted by the one-step RT-PCR kit (Qiagen), and second-round PCR was conducted by the Platinum high-fidelity Taq DNA polymerase (Life Technologies, USA). After purification with the QIAquick PCR purification kit (Qiagen), all PCR products were directly sequenced on an ABI 3730 Sanger-based genetic analyzer, and the genome was assembled using DNAStar version 5.0.

The complete genome sequence of this new isolate, GZ37, is 1,680 nucleotides (nt) in length. Phylogenetic analysis based on the nucleic acid sequence of the complete genome was conducted using the neighbor-joining method. GZ37 belongs to genotype 1, together with most Middle Eastern and Southeast Asian strains. Another Chinese HDV strain, JN, also belongs to genotype 1 but clusters with European and African strains. GZ37 has a close relationship with HDV-1 isolates from Turkey (dTk34, accession no. AM779588), Iran (D7-D, accession no. KJ744223), and Taiwan (TW1435#47, accession no. AY648956). The nucleotide similarities to these strains are 95.1%, 93.9%, and 91.9%, respectively, and its nucleotide similarity to strain JN is 91.4%. This epidemic of HDV strains in Guangdong was possibly initiated by the introduction of HDV from the Middle East.

Our previous study indicated that the prevalence of HBV/HDV coinfection in Guangdong is not low, at about 6.5% (8). The complete genome sequence of the Chinese HDV-1 strain described here will help us understand the molecular epidemiology and contribute to the control of HDV infections in China in the future.

### Nucleotide sequence accession number.

The complete genome sequence of HDV-1 strain GZ37 has been submitted to GenBank under accession number KT722840. The version described in this paper is the first version.
